# Molecular networking derived from untargeted LC-MS/MS analysis to discover inhibitors of RANKL-induced osteoclastogenesis from Egyptian marine sponge-associated fungi

**DOI:** 10.1038/s41598-025-12456-y

**Published:** 2025-07-25

**Authors:** Abdelhalim A. Elgahamy, Ahmed H. El-Desoky, Asmaa M. Otify, Ahlam M. El Fishawy, Ahmed A. El-Beih

**Affiliations:** 1 Chemistry of Natural and Microbial Products Department, National Research Centre, 33 El-Bohouth St., Dokki, Giza, 12622 Egypt; 2 Pharmacognosy Department, National Research Centre, 33 El-Bohouth St, Dokki, Giza, 12622 Egypt; 3 Pharmacognosy Department, Faculty of Pharmacy, Cairo University, Kasr- El-Ainy, Cairo, 11562 Egypt

**Keywords:** Bisphenols, Osteoclastogenesis, *Aspergillus flavus*, Metabolomics, *Cladosporium*, Chemical biology, Drug discovery

## Abstract

In continuous search for RANKL induced osteoclastogenesis inhibitors, twenty-six fungal isolates were obtained from ten Red Sea marine sponges collected from Egypt and the ethyl acetate fractions of their cultures’ methanol extracts were assessed in RAW264 macrophages. Active fractions were profiled *via* LC-MS/MS, followed by untargeted molecular networking, leading to the tentative identification of eight unreported compounds (**1**, **A2**, **C1**,** C2**,** C4**-**C7**), and thirteen known compounds. The two active fungi were identified and deposited in GenBank with accession numbers PQ423742 and PQ423748 for *Aspergillus flavus* and *Cladosporium colombiae*, respectively. Bioassay-guided isolation afforded two bisphenol diglycidic ethers, **1** and **2**, and two diketopiperazines, **3** and **4** from *A. flavus*, while *C. colombiae* yielded cinnamic acid (**5**), two diketopiperazines (**6** and **7**), and altenuene (**8**). Structures were elucidated by NMR and mass spectroscopic analyses, which revealed **1** to be isolated for the first time from natural sources. Isolated compounds were biologically evaluated. Only **1** and **8** inhibited RANKL-induced formation of mature multinucleated osteoclasts with IC_50_ 57.14 and 38.35 µM, respectively, without cytotoxicity against RAW264 macrophages. The ADMET properties for **1** and **8** were predicated using SwissADME and pkCMS platforms. **8** showed superior solubility and lower toxicity than compound **1**.

**Supplementary Information:**

The online version contains supplementary material available at 10.1038/s41598-025-12456-y.

## Introduction

Osteoporosis is a global health problem with high incidence among geriatric populations. This silent disease has high global preference, characterized by disturbance in osteoblast-osteoclast coupling mechanisms^1,2^. Osteoclastogenesis, the process by which osteoclasts are formed, is initiated when the monocyte/macrophage lineage is activated by receptor activator of nuclear factor-κB ligand (RANKL). Upon RANKL stimulation, downstream signaling pathways, including NF-κB and Mitogen-activated protein kinase are activated, leading to the upregulation of osteoclast-specific genes such as tartrate-resistant acid phosphatase (TRAP) and fusion-related enzymes. Dysregulation of osteoclast functions has been implicated in various diseases like osteoporosis and bone metastasis^3^. Overactivation of osteoclasts leads to excessive bone resorption, thereby reducing bone mineral density^4^. It is predicted that, by 2050 osteoporotic patients will be around 200 million worldwide and the economic toll will exceed 130 billion USD^5^. Therefore, global attention was directed to finding alternative sources for inhibiting osteoclastogenesis with potential therapeutic intervention^6–8^.

Fungal metabolites are structurally diverse and show wide spectrum of biological activities. Being easily cultivated, fungi are considered as renewable promising source to overcome the limitation of the short supply, that is a main concern in other natural resources such as plants and marine invertebrates. *Aspergillus* is a prolific fungal Genus able to produce a wide range of secondary metabolites. That includes alkaloids such as the antibacterial compounds pseurotin A, fumitremorgin C, and bisdethiobis(methylthio)gliotoxin, from *A. terreus*^9^antiosteoclastogenic glycosides such as taichunins J and K from *A. taichungensis*^14^peptides such as the antibacterial and cytotoxic malformin C from *A. niger*^10^polyketides such as calidiol A from *A. californicus* that exhibited moderate antibacterial activity against MRSA^11^and immunosuppressive steroids such as Aspersteroids from *A. ustus*^12^. These metabolites exhibit a broad spectrum of biological activities with significant medical, industrial, agricultural, and economic importance^13^. *Aspergillus* sp. metabolites have shown considerable biological activities against osteoclast hyperactivity such as taichunins with inhibitory activity on RANKL induced osteoclastogenesis^14^.

Fungal secondary metabolites possess diverse bioactivities and can modulate bone remodeling through multiple mechanisms, including antioxidant, anti-inflammatory, and gene-targeting effects relevant to osteoclast differentiation and function^6,15^. Despite their therapeutic potential, few studies have systematically explored their role in regulating osteoclastogenesis. In this study, we employed murine RAW264 macrophages as a validated in vitro model to identify fungal-derived inhibitors of osteoclast differentiation. This strategic approach not only facilitates the discovery of new bioactive scaffolds but also addresses a critical unmet need for alternative osteoclast-targeted therapies capable of restoring bone homeostasis in osteoporosis and other bone-resorptive disorders, while avoiding the adverse effects of conventional treatments such as bone osteolysis of bisphosphonates^16^osteonecrosis of jaw, hypocalcemia and atypical femur fractures in case of denosumab^17^and cardiovascular, musculoskeletal adverse effects of parathyroid hormone (PTH) analogs, such as teriparatide and abaloparatide^18^. Meanwhile, SwissADME and pkCSM Web Servers were used for ADMET Evaluation of the active compounds for predicting their pharmacokinetic and safety profiles^19^.

## Materials and methods

### General experimental procedure

Optical rotations were recorded on JASCO DIP-1000 polarimeter in methanol (CH_3_OH)^1^. H- and ^13^C-NMR spectra were measured at field strengths 600 and 150 MHz, respectively, by Bruker Avance III 600 NMR spectrometer in deuterated chloroform-*d* (CDCl_3_) and methanol-*d*_4_ (CD_3_OD). Chemical shifts were referenced to the residual solvent peaks (*δ*_H_ 7.26 and *δ*_C_ 77.0, *δ*_H_ 3.31 and *δ*_C_ 49.0 for CDCl_3_ and CD_3_OD, respectively). Mass spectra were recorded on a Bruker ESI-ion trap amaZon speed system. MPLC was carried out by Biotage Isolera I system, equipped with a UV detector, while HPLC purifications were conducted on Waters 515 HPLC pump, connected to Waters 2489 UV/visible detector equipped with Pantos Unicorder U-228.

All solvents of extraction, fractionation, column chromatography, and MPLC were of analytical grade (Alpha Chemika, India), while solvents for LC-MS and HPLC purifications were of HPLC grade (Fischer, India). All salts for buffers, media constituents, and standard, as well as chemicals and solvents in the TRAP activity assay, were of fine grade (Sigma, Aldrich, India).

### Isolation and identification of the endophytic fungal isolates

Samples of ten sponges (Table S1) were collected from Magawish Island at N 27.169714, E 33.833056 using SCUBA equipment at around 7 m depth, Red Sea, Egypt, and identified as previously reported^20^. The isolated fungi (Figure S1) were identified on the basis of their morphological features of fungal culture and hyphae (Figure S3). The identification of the two selected fungi, *Aspergillus flavus* (*A. flavus*) and *Cladosporium colombiae* (*C. colombiae*) isolated from *Ircinia variabilis*, and *Hyrtios erectus*, respectively (Figure S2), were confirmed by sequence analysis and data comparison from NCBI GenBank databases (Figure S4). These fungi were assigned the accession numbers PQ423742 for *A. flavus* and PQ423748 for *C. colombiae*^21–23^.

### Fungal cultivation, extraction, and fractionation for screening purposes

The isolated fungi were cultured on biomalt-peptone agar medium (g/L) (biomalt 20, peptone 12.5, agar 20, seawater 50%), two plates/isolate. All cultures were incubated for 30 days at 28 °C. All solid media were extracted with CH_3_OH 100 mL/plate three successive times and filtered on Whatman filter paper No.1. Extracts were concentrated under vacuum at 40 °C and the resulting aqueous gummy residues were resuspended in water and partitioned with ethyl acetate (EtOAc). The obtained fractions were kept in dim light containers for further biological and chemical screening.

### Evaluation of the antiosteoporosis activity

#### Cell culture and RANKL-induced osteoclastogenesis

EtOAc fractions of all fungal CH_3_OH extracts were tested for their ability to inhibit TRAP activity and the formation of multinucleated osteoclasts (MNCs) in RANKL-treated RAW264 macrophages as previously reported^7,24^. Briefly, RAW264 cells (RIKEN Cell Bank, Tsukuba) were cultured in MEMα medium supplemented with 10% heat-inactivated fetal bovine serum and 1% penicillin/streptomycin and incubated at 37 °C in a 5% CO_2_ humidified atmosphere in two 96-well plates at a density of 1000 and 6000 cells/well, for TRAP assay and MNCs staining assay, respectively. Samples were then added at a concentration of 50 and 20 µg/mL for extracts, 50, 20, and 10 µM for pure compounds, and 83 µM for the standard quercetin. Then, soluble RANKL (Santa Cruz, USA) was added at a concentration of 50 ng/mL to all wells except for negative control wells. Dimethyl sulfoxide was used as a vehicle in both negative and positive control wells.

#### TRAP activity assay

After four days, cells were washed with phosphate-buffered saline (PBS) and lysed with lysis buffer formed of 50 mM sodium tartrate, 50 mM sodium acetate, 150 mM potassium chloride, 0.1% Triton X-100, 1 mM sodium ascorbate, and 0.1 mM ferric chloride, pH 5.7; 100 µL/well on ice for 10 min. The resulting cell extract (20 µL) was added to 100 µL of TRAP buffer containing 2.5 mM *p*-nitrophenyl phosphate (Thermo Fisher Scientific) and incubated at 37 °C for 4 h. To stop the reaction, 50 µL of 0.9 M sodium hydroxide was added to the reaction mixture and the reaction product (*p*-nitrophenolate) was measured as the absorbance at 405 nm.

#### Multinucleated TRAP + ve osteoclast (MNCs) counting assay

Following a 4-day incubation period, the differentiated cells were fixed with 4% paraformaldehyde solution (Sigma–Aldrich, USA), washed with PBS, and stained using a TRAP-staining solution, which consisted of 50 mM sodium tartrate, 45 mM sodium acetate, 0.1 mg/mL naphthol AS-MX phosphate (Sigma– Aldrich, USA), and 0.6 mg/mL fast red violet LB salt (Sigma–Aldrich, USA), pH 5.2, for 1 h or longer at room temperature. TRAP-positive cells, that stained red and contained 3 or more nuclei were determined to be multinuclear osteoclasts and were quantified in each well by counting MNCs in 8 fields/well using a light microscope^8^.

#### MTT cytotoxicity assay

After incubation of cells for one day, media was aspired and replaced with new one supplemented with treatments, whereas controls contained only DMSO. Cells were then kept at 37 °C for three days. Finally, 50 µL of 3-(4,5-dimethylthiazol-2-yl)−2,diphenyltetrazolium bromide (MTT) solution (150 µg/mL in medium) was added after aspiration of medium, incubated under the same conditions for 3 h. Then 200 µL of DMSO were added to each well, after removal of the reaction solution, to dissolve the formed formazan salt crystals, and optical density was measured at 570 nm with a microplate reader.

In all biological tests, experiments were performed in triplicates, results were expressed as mean ± standard deviation, and significant differences were set at *p* < 0.005 according to the Student’s t-test.

### LC-MS/MS analysis and metabolic molecular network

#### LC-MS/MS conditions and parameters

To prepare the samples, 1 mg of each EtOAc fraction was dissolved in 1 mL of CH_3_OH (HPLC grade, Fisher, UK) and filtered through a 0.22 μm PTFE membrane (Sartorius, USA). The HPLC analysis was performed using a Shimadzu Prominence system, featuring LC-20AD pumps and an SPD-M20A DAD detector, coupled with a Bruker amaZon speed mass spectrometer. The system operated in both positive and negative ion modes within the same run. Background subtraction based on blank mobile phase run was carried out.

For each analysis, 1 µL of the prepared extract was injected into an InertSustain-C18 column (3.0 μm, 4.6 × 150 mm; GL Sciences, Japan). The elution was achieved using a gradient with two solvents: solvent A (100% water + 0.1% acetic acid) and solvent B (100% acetonitrile + 0.1% acetic acid). The gradient program was set as follows: starting with 10% B for the first minute, increasing linearly from 10 to 100% B over the next 14 min, at a flow rate of 0.4 mL/min.

The ESI source conditions included a capillary temperature of 320 °C, a source voltage of 3.5 kV, and a sheath gas flow rate of 11 L/min. Ion detection was conducted in full scan mode with range 100–2000 *m/z*, MS(n) ICC target and MS(n) maximum acquisition time were adjusted to 50,000 and 200 ms, respectively, with a threshold of 1000 counts and a scan rate of 6 scans per second^25^.

The MS/MS data were converted from Bruker’s “.d” files to mzML format using MSConvert, part of the ProteoWizard suite (https://www.proteowizard.org). The processed data were then uploaded to the Global Natural Products Social Molecular Networking (GNPS) platform for further analysis.

#### Molecular network

Molecular networks for two EtOAc fractions were created using the GNPS platform (http://gnps.ucsd.edu). In accordance with the default GNPS networking parameters, a mass tolerance of 0.2 Da was applied for precursor ions and 0.05 Da for fragment ions. For network creation, a minimum of six matching MS fragments between two consensus MS/MS spectra was required, and the similarity threshold (cosine score) for connecting nodes was set at 0.7. The resulting network was saved as a GraphML file and visualized using Cytoscape 3.7.1 software, employing a force-directed layout.

#### Large-scale culture and extraction

*A. flavus* and *C. colombiae* were selected based on bioassay results and cultured on biomalt-peptone agar medium (g/L) to fill 160 petri dishes (100 mm x 15 mm) for each fungus (25 mL medium/petri dish). After inoculation (30 days/28 °C), each fungal culture was extracted with CH_3_OH, filtrated, and concentrated under pressure (40 °C). The obtained residues were dissolved in water (H_2_O), partitioned with EtOAc to be concentrated using Rota evaporator, and afforded 580 and 586 mg of EtOAc fractions for *A. flavus* and *C. colombiae*, respectively.

#### Isolation of fungal metabolites

For *A. flavus*, EtOAc fraction (580 mg) was processed using MPLC (SNAP Ultra^®^, 10 g, Biotage Japan Ltd.) with a gradient system (0–10% CH_3_OH/CH_2_Cl_2_ for 8 column volumes, then, 10–100% CH_3_OH/CH_2_Cl_2_ for 5 column volumes, and finally washed with CH_3_OH for 5 column volumes at 36 mL/min) to produce six fractions (Frs. V1-V6). Fr. V5 (99.1 mg) underwent further separation using octadecylsilane (ODS) reverse-phase open column chromatography using gradient elution systems of CH_3_OH/H_2_O at 40%, 70%, and 100% resulting in six subfractions (Frs. X1-X6). Fr. X3 (16.1 mg) was subsequently purified by HPLC (Inertsil ODS-P column, 20 × 250 mm) with 70% CH_3_OH/water to isolate compounds **1** (4.5 mg) and **2** (1.3 mg). Fr. V2 (45.2 mg) was refined through preparative HPLC (Inertsil Diol, GL Sciences Inc., 20 × 250 mm) eluted with CH_2_Cl_2_/CH_3_OH 50:1 to obtain compounds **3** (11.5 mg) and **4** (4.6 mg).

EtOAc fraction of *C. colombiae* (586 mg) was manipulated by MPLC (SNAP Ultra^®^, 25 g, Biotage Japan Ltd.) with a gradient system (0–5% CH_3_OH/CH_2_Cl_2_ for 13 column volumes, then, 5-100% CH_3_OH/CH_2_Cl_2_ for 3.5 column volumes, and finally washed with CH_3_OH for 3.5 column volumes at 75 mL/min) to give six fractions (Frs. Y1-Y6). Fr. Y3 (8.88 mg) was purified via ODS HPLC (Inertsil ODS-P column, 20 × 250 mm) with 50% CH_3_OH/H_2_O to provide compounds **5** (9.9 mg), **6** (5.38 mg), and **7** (2.36 mg). Fr. Y5 (110.47 mg) was loaded to an ODS open column, eluted with 50% CH_3_OH/H_2_O then CH_3_OH, resulting in two subfractions (Frs. Z1 and Z2). Upon purification of Fr. Z1 (34.18 mg) by ODS HPLC (Inertsil ODS-P column, 20 × 250 mm) with 50% CH_3_OH/H_2_O, **8** (5.90 mg) was obtained.

#### Prediction of ADMET parameters

For active compounds, the physicochemical and drug-likeness characters were obtained using SwissADME web server (http://www.swissadme.ch/), while ADMET properties were predicated using pkCMS web server (http://biosig.unimelb.edu.au/pkcsm/prediction). These analyses provided insights into essential parameters in early drug development^26^.

## Results and discussion

### LC-MS/MS analysis and metabolic molecular network

**Fig. 1:**
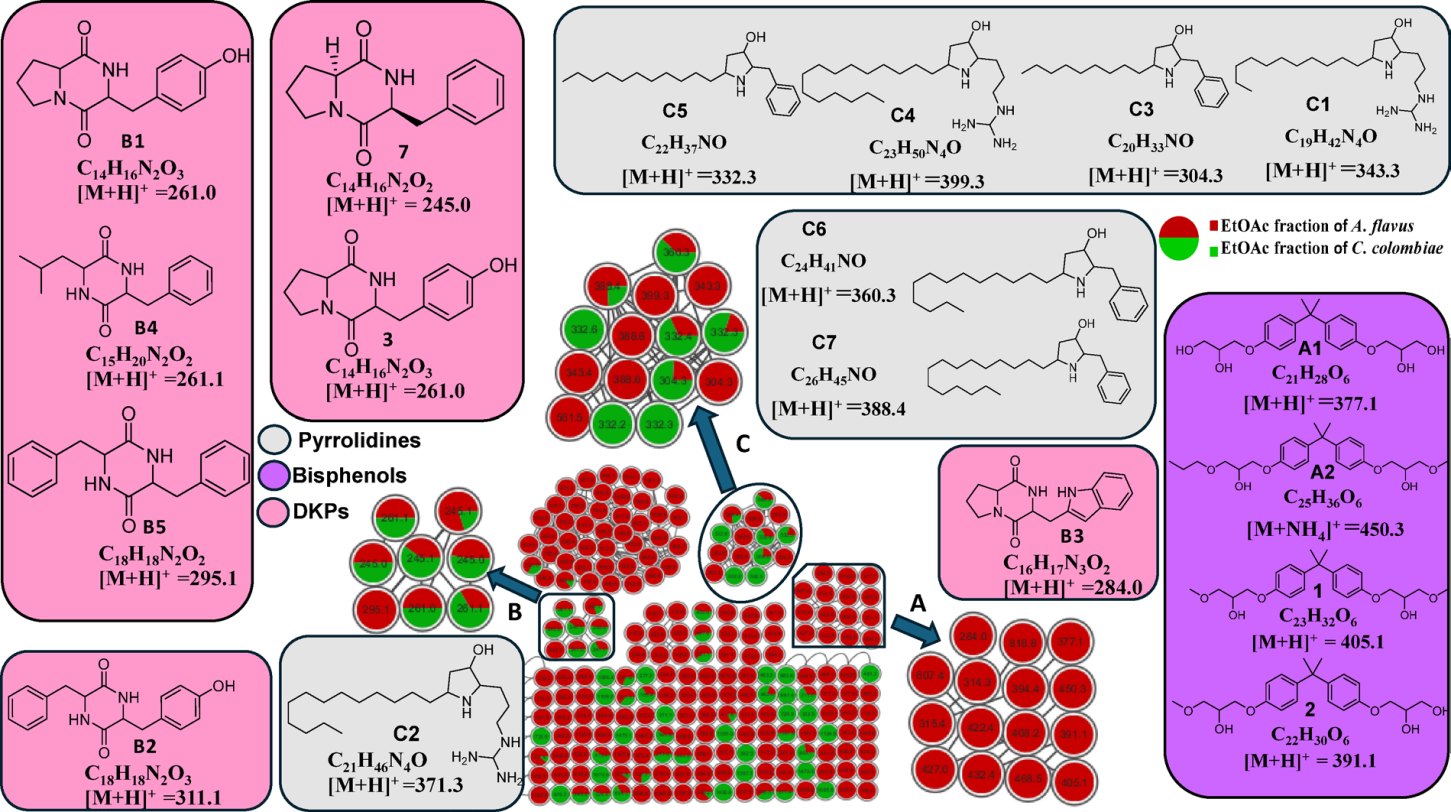
Molecular network established using LC-MS/MS data of the EtOAc fractions of *A. flavus* (red) and *C. colombiae* (green) in the positive ion mode. Each node is displayed as a pie chart to reflect the relative abundance of each molecular ion in the analyzed samples. Cluster A: bisphenols, cluster B: DKPs, and cluster C: pyrrolidine alkaloids.

Ten fungal species were isolated from ten Red Sea Egyptian marine sponges (Table S1, Figure S1). Their EtOAc fractions, obtained from the crude CH_3_OH extracts of corresponding cultures, were tested for their ability to inhibit the formation of osteoclasts from RANKL-treated RAW264 macrophages. The EtOAc fractions of two fungal isolate cultures significantly exhibited potent antiosteoporosis activity, as they completely inhibited the TRAP activity in RANKL-stimulated RAW264 cells at 50 µg/mL, with an IC_50_ of 24.83 µg/mL for *Aspergillus flavus* and 17.18 µg/mL for *Cladosporium colombiae*. Metabolomic profiles of the two bioactive fractions were monitored by LC-MS/MS analysis (Figure S5a, Table S2), followed by untargeted molecular networking for dereplication of the secondary metabolites within the chemical space of each extract (Fig. 1). This process resulted in the tentative identification of eighteen compounds, which are primarily classified as bisphenols, diketopiperazines (DKPs), and pyrrolidine alkaloids.

#### Bisphenols

The generated molecular networks of *A. flavus* extract (Fig. 1) revealed a distinct cluster (A) of bisphenol compounds characterized by a bisphenol diglycidic ether structure. In detail, the node at *m/z* 377.1 corresponds to 2,2-bis‐[4‐(2,3‐dihydroxy-propoxy)phenyl]propane (compound **A1**), exhibiting an MS/MS spectrum (Figure S5b) with a base peak at *m/z* 209 resulting from the characteristic loss of 168 amu, attributed to a glyceryl phenol moiety^27^. Similarly, compound **2** (*m/z* 391.1) shows a similar fragmentation pattern, featuring a neutral loss of 182 amu corresponding to a methyl glyceryl phenol moiety, resulting in a base peak at *m/z* 223, and was identified as 2‐[4‐(2-hydroxy-3‐methoxy-propoxy) phenyl]‐2‐[4‐(2,3-dihydroxy propoxy) phenyl] propane (Fig. 1, S5b). Consequently, compounds **1** (*m/z* 405.31) and **A2** (*m/z* 450.53 as ammonia adduct) were determined to be 2,2‐bis‐[4‐(2‐hydroxy-3-methoxy-propoxy)phenyl]propane, and 2-[4-(2-hydroxy-3-methoxy-propoxy)phenyl]−2-[4-(2-hydroxy-3-propoxy-propoxy)phenyl]propane (Fig. 1, S5b).

#### Diketopiperazines (DKPs)

The fragmentation patterns of diketopiperazines (DKPs) (Table S2, Figure S5c) provide critical insights into their structural identification, particularly through the detection of specific ions such as *m/z* 120, corresponding to the phenylalanyl moiety, and *m/z* 136, corresponding to the tyrosyl moiety. By analyzing these ions alongside the *m/z* values and molecular formulas of the parent ions, the two amino acids constituting the DKP compounds can be deduced. For example, the parent ion with *m/z* 261.0 (C_14_H_16_N_2_O_3_, Rt 1.9) exhibits a fragment at *m/z* 136, indicating the presence of a tyrosyl moiety, suggesting the structure is cyclo(prolyl-tyrosyl) (**B1**)^28^. Similarly, the parent ion with *m/z* 311.1 (C_18_H_18_N2O_3_) shows fragments at *m/z* 136 (tyrosyl) and *m/z* 120 (phenylalanyl), pointing to the structure cyclo(phenylalanyl-tyrosyl) (**B2**)^28^.

Other observed fragments result from the loss of a carbonyl group (CO, −28), as seen in the fragmentation patterns. For instance, the parent ion with *m/z* 295.1 (C_18_H_18_N_2_O_2_) loses a CO group to yield a fragment at *m/z* 267, consistent with the structure cyclo(phenylalanyl-phenylalanyl) (**B5**)^29^. Similarly, the parent ions with *m/z* 261.0 (C_14_H_16_N_2_O_3_, Rt 2.1) and *m/z* 245.0 (C_14_H_16_N_2_O_2_) lose CO to produce fragments at *m/z* 233 and 216, respectively, indicating the structures cyclo(hydroxyprolyl-phenylalanyl) (**3**) and cyclo(prolyl-phenylalanyl) (**7**)^30,31^. Additionally, the parent ion with *m/z* 261.1 (C_15_H_20_N_2_O_2_, Rt 4.6) shows fragments at *m/z* 120 (phenylalanyl) and 233, suggesting the structure cyclo(leucyl-phenylalanyl) **(B4)**^29^. These patterns highlight how the loss of CO, along with the detection of specific ions, aids in the structural elucidation of DKPs.

#### Pyrrolidine alkaloids

The molecular network (Fig. 1, cluster C) showed various nodes with parent ions at *m/z* 304.3 (compound **C3**), 332.3 (**C5**), 360.3 (**C6**), and 388.4 (**C7**), and base peaks at *m/z* 212, 240, 268 and 296, respectively (Figure S5d, Table S2). These resulted from the neutral loss of a benzyl moiety (92 amu), and suggestive of benzyl-hydroxy-pyrrolidine moiety with a long alkyl substituent^32^. Notably, all the aforementioned nodes exhibit a 28 amu increase, both in the parent and fragment ions after the neutral loss of the benzyl moiety. This pattern suggests that each compound is two methylenes larger than the previous one, and their structures have been proposed as 2-benzyl-3-hydroxy-5-nonyl-pyrrolidine (**C3**), 2-benzyl-3-hydroxy-5-undecyl-pyrrolidine (**C5**), 2-benzyl-3-hydroxy-5-tridecyl-pyrrolidine (**C6**), and 2-benzyl-3-hydroxy-5-pentadecyl-pyrrolidine (**C7**).

**Fig. 2:**
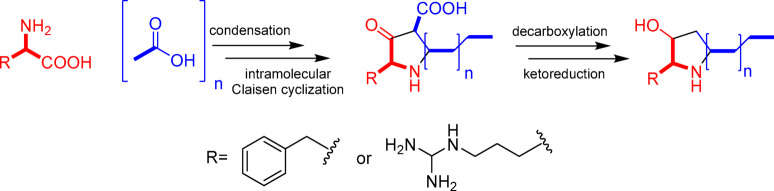
Suggested biosynthetic pathway of the detected pyrrolidine alkaloids.

Fukuda and co-workers revealed the biosynthetic pathway of preussin B, a similar alkyl-hydroxy-pyrrolidine metabolite^33^. They proved that such metabolites were formed by the condensation of phenylalanine amino acid with a polyketide chain, followed by Claisen-type intramolecular cyclization, reduction, and malonate-based side chain elongation (Fig. 2). Such metabolic pathway explains the two-carbon extension pattern of metabolites. Furthermore, the incorporation of amino acids in the biosynthetic pathway (Fig. 2) explains the structures of other nodes displaying parent ions at *m/z* 343.3 and 399.3 in the same cluster (C), with base peaks at *m/z* 240 and 296, respectively. These fragment ions result from the neutral loss of 103 amu, corresponding to the side chain of the amino acid arginine (Figure S5d). Consequently, the structures of such compounds could be tentatively suggested as 2-(3-diaminomethyl-aminopropyl)−3-hydroxy-5-undecyl-pyrrolidine (**C1**), and 2-(3-diaminomethyl-aminopropyl)−3-hydroxy-5-pentadecyl-pyrrolidine (**C4**) (Figure S5d, Table S2). Apart from cluster C, a singleton node (*m/z* 371.3) exhibited similar fragmentation patterns as previously discussed and was annotated as 2-(3-diaminomethyl-aminopropyl)−3-hydroxy-5-tridecyl-pyrrolidine (**C2**), respectively (Fig. 1).

The proposed structures of **1**, **A2**,** C1-C2**,** and C4-C7** were suggested based on available MS/MS data and biosynthetic similarities in other fungal species, have not yet been reported in natural products literature to date and require further isolation and detailed structure elucidation for the confirmation of their structures and configurations.

### Isolation of fungal metabolites

EtOAc fractions of the selected fungal extracts were subjected to bioassay-guided purification leading to the isolation of a new natural metabolite **1**, alongside seven previously known compounds **2**–**8**. Notably, four of these compounds were detected in the LC-MS/MS analysis conducted in this study (Fig. 1, Table S2). The aim of the isolation process is to identify and characterize the key bioactive compounds within the bioactive EtOAc fractions of the two fungi, which may be responsible for the observed antiosteoporosis activity, thereby facilitating the development of targeted therapeutic agents.

Compound **1** was isolated as an optically inactive colorless solid $\:{\left[{\upalpha\:}\right]}_{\text{D}}^{25}$ +0.01 (*c* 2.7, MeOH), its molecular formula C_23_H_32_O_6_, was deduced from HRESI-MS *m/z* 427.2095 [M + Na]^+^ calculated for C_23_H_32_O_6_Na.

^13^C-NMR spectrum (Table 1, Figure S6b) showed only 10 resonances indicating the presence of overlapped signals^1^. H-NMR (Table 1, Figure S6a) and HSQC (Figure S6d) spectra revealed the presence of six protons’ singlet [*δ*_H_ 1.62 (s), *δ*_C_ 32.0, (C-6 and 6’)], two singlet methoxy groups, four ortho-coupled methines, four oxymethylenes, and two oxymethines.

Molecular formula together with signal integration and chemical shift distribution provided evidence that **1** is dimeric in nature. HMBC (Table 1, Figure S6e) from H-2, H-2’, H-3, and H3’ to C-1 and C-4 together with the chemical and magnetic equivalence of H-2/H-2’ and H-3/H-3’ established the *p*-substituted ring system of **1**. The chemical shift of C-1 at *δ*_C_ 156.3 suggested the phenolic nature of C-1. On the other hand, COSY correlations (Figure S6c) established the spin system H-7/H-8/H-9 of the glycerol moiety, and the HMBC cross peak from H_2_−7 to C-1 confirmed the connection of the glyceryl moiety to C-1, while the HMBC from H_3_−10 to C-9 suggested the methoxylation of C-9.

The methyl singlet H_3_−6 (*δ*_H_ 1.62) showed HMBC to the aliphatic quaternary carbon (*δ*_C_ 41.66, C-5), and the aromatic quaternary carbons (*δ*_C_ 143.50, C-4). This confirmed the *para* substitution of the bisphenol *via* C-5. The methyl singlet also showed an HMBC correlation to an attached carbon, indicating the overlap of gem-dimethyl groups. The aliphatic moiety at C-1 was confirmed by HMBC correlations of methylene protons (*δ*_H_ 3.99) to C-1, and the other methylene carbon (*δ*_C_ 73.44, C-9). Moreover, methylene protons (*δ*_H_ 3.56) showed HMBC correlations to oxy methine (*δ*_C_ 68.80, C-8), and oxy methyl (*δ*_C_ 59.24, C-10). On the basis of these data, the structure was confirmed as a dimeric compound, and by comparing the obtained data with the available literature, compound **1** (Fig. 4; Table 1) was assigned 2,2-bis‐[4‐(2‐hydroxy-3-methoxy-propoxy)phenyl]propane, characterized by dimethyl substitution at the terminal hydroxyl groups on both sides, unlike the other bisphenol derivatives. To the best of our knowledge, this is the first report of this compound as a naturally occurring metabolite.

Compound **2** (Fig. 4) was isolated as a colorless solid whose chemical formula C_22_H_30_O_6_ was deduced from ESI-MS *m/z* 413.34 [M + Na]^+1^. H-NMR and ^13^C-NMR spectra (Figures S7a, S7b) were very similar to that of **1** and matched with the reported spectra of 2-[4‐(2-hydroxy-3-methoxy-propoxy)phenyl]‐2‐[4‐(2,3-dihydroxy-propoxy)phenyl]propane^34^.

**Fig. 3:**
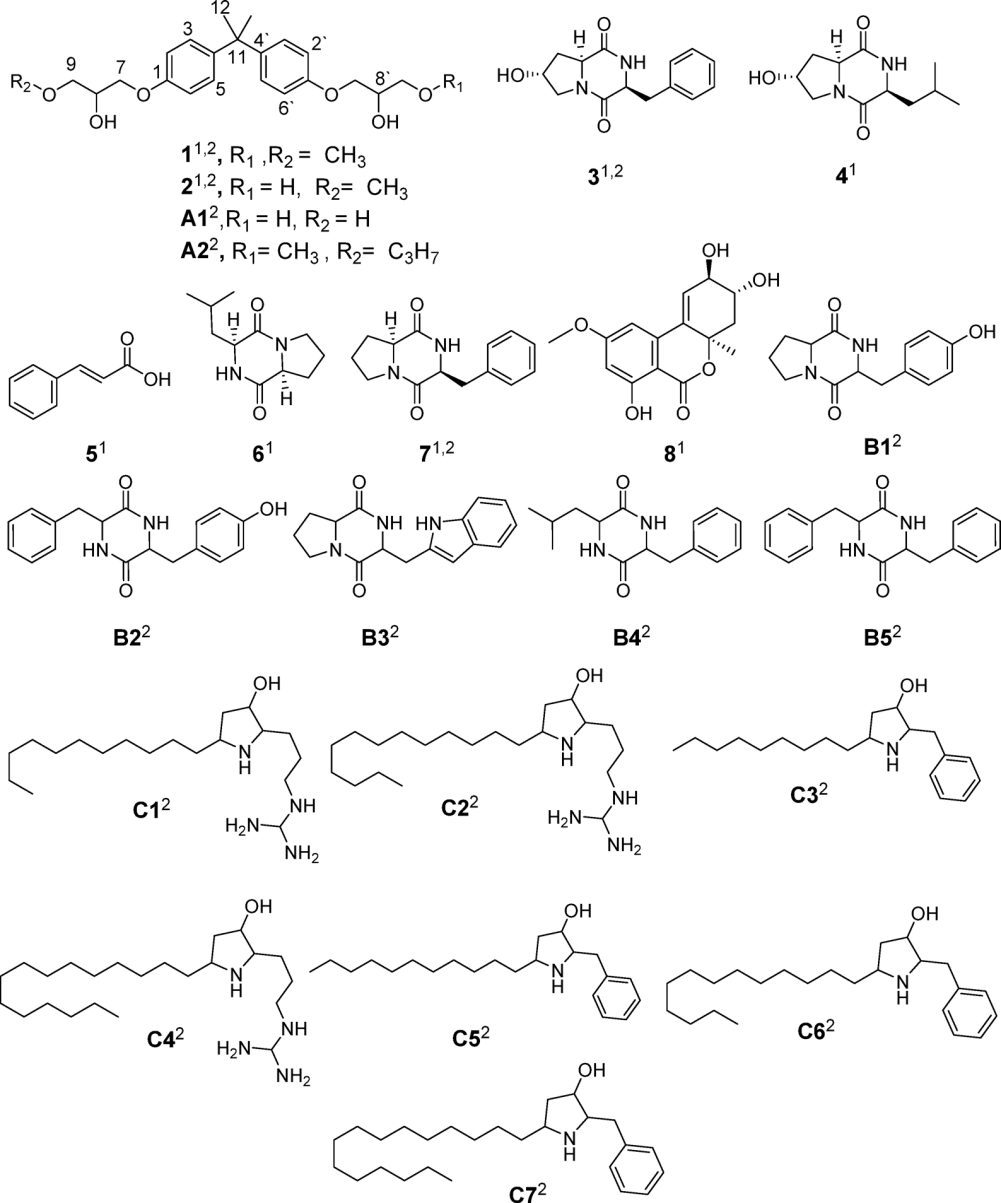
Structures of compounds of *A. flavus* and *C. colombiae* EtOAc fractions^1^ isolated pure compound^2^, detected by LC-MS/MS).

**Fig. 4:**
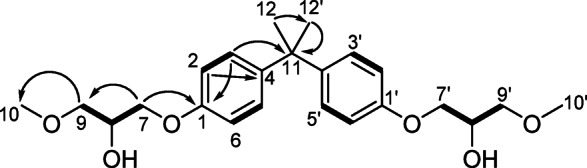
^1^H-^1^H COSY (bold lines) and key HMBCs (arrows) of compound **1**.

**Table 1:** ^1^H-NMR, ^13^C-NMR, and HMBC assignments of **1** in CDCl_3_ (*δ* in ppm).

No	δ_C,_ type	δ_H_ Multiplicity (J in Hz)	HMBC
1, 1’	156.3, C		
2, 2’, 6, 6’	113.9, CH	6.81, d (8.8)	1, 4, 6
3, 3’, 5, 5’	127.7, CH	7.13, d (8.8)	1, 2, 3, 11
4, 4’	143.5, C		
7a, 7’a	69.0, CH_2_	3.98, dd (9.4, 6.0)	1, 8, 9
7b, 7’b		4.01, dd (9.4, 4.4)	
8, 8’	68.8, CH	4.14, m	7, 9
9a, 9’a	73.4, CH_2_	3.53, dd (9.7, 6.0)	7, 10
9b, 9b’		3.58. dd (9.7, 4.4)	
10, 10’	59.2, CH_3_	3.40, s	9
11, 11’	41.7, C		
12, 12’	32.0, CH_3_	1.62, s	4, 11, 12’
8-OH, 8’-OH		5.29, s	

Other isolated compounds (Fig. 4) were identified by comparison of their spectral data with previously reported compounds and were revealed to be 3S*,6S*,8R*-cyclo(*trans*−8-hydroxyprolyl-phenylalanyl) (3)^30,31^3S*,6S*,8R*-cyclo(*trans*−8-hydroxyprolyl-leucyl) (**4**)^35^*trans*-cinnamic acid (**5**)^36^3S*,6S*-cyclo(prolyl-leucyl) (**6**)^35^3S*,6S*-cyclo(prolyl-phenylalanyl) (**7**)^35^and altenuene (**8**)^37^ (Figs. 4, S8).

### Evaluation of the antiosteoporosis activity of the isolated compounds

**Fig. 5:**
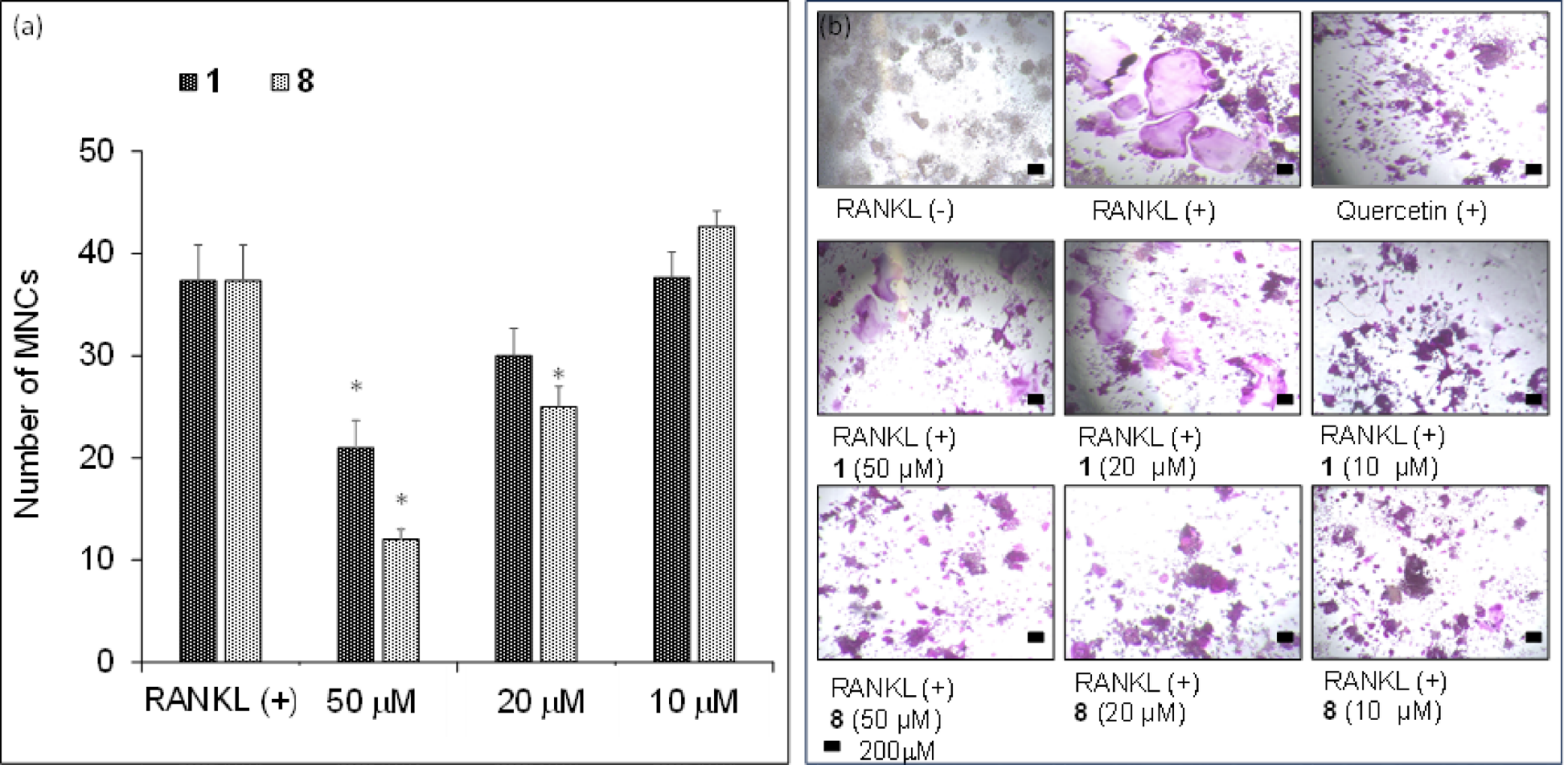
(**a**) Effects of compounds **1** and **8** on RANKL-induced osteoclast differentiation of RAW 264 cells. RAW 264 cells were treated with RANKL (50 ng/mL) in the presence or absence of **1** or **8** at the indicated concentrations and cultured for 4 days. The cells were stained using a TRAP-staining solution, and the number of TRAP-positive multinuclear (> 3 nuclei) cells was counted. Experiments were performed in triplicate, error bars stand for standard deviation, and asterisks show significant differences at *p* < 0.005 according to the Student’s t-test. (**b**) TRAP-stained RAW 264 cells incubated with 50 ng/mL RANKL in the presence or absence of **2** or **8** (20 µM). The Scale bar represents 200 nm.

All isolated compounds were tested for their ability to inhibit osteoclastogenesis upon stimulation of RANKL-induced differentiation of RAW264 cells with the formation of MNCs. Compounds **1** (2,2-bis‐[4‐(2‐hydroxy-3-methoxy-propoxy)phenyl]propane) and **8** (altenuene) significantly inhibited the formation of MNCs, with IC_50_ values 57.14 and 38.35 µM, respectively, with no toxic effect against RAW264 macrophages at the highest tested concentration (Fig. 5). Other compounds showed no inhibition against RANKL-induced formation of MNCs from RAW264 macrophages.

Compound **1** has the bisphenol skeleton, which is related to bisphenol A as the simplest member of such structural family. Some studies discussed the effect of bisphenol A on bone metabolism as it mimics endogenous estrogen, inhibits osteoblast, and leads to bone loss^38,39^. Bisphenol compounds exert both estrogenic agonism, *via* binding to ERα and ERβ and antiandrogenic activity. Both properties that can influence bone remodeling. For example, BPAF binds ERα with nanomolar affinity and functions as a full agonist, upregulating genes such as *Runx2* and *osteocalcin*, which promote osteoblast differentiation and bone formation^40^. Conversely, these compounds antagonize the androgen receptor (AR), blocking AR-mediated transcriptional programs that normally support osteoblast proliferation and inhibit osteoclastogenesis^41^. Because androgens, and AR signaling contribute to bone mass maintenance in both sexes, antiandrogenic bisphenols may counterbalance their estrogenic benefits, especially in men or premenopausal women.

As the aliphatic chain substituting the glyceryl residue increases, the amelioration of dexamethasone-induced osteoporosis is increased, such as in the case of bisphenol A diglycidyl ether^42^. Such observation supports the difference in activity between **1** and **2**, where increasing the degree of methylation in **1** rendered it more active than **2**. This observation can offer an additional perspective for developing more active semisynthetic derivatives of bisphenol diglycidic acid ethers based on the structure-activity relationship deduced from **1** to **2**.

On the other hand, **8** isolated from *C. colombiae* exhibited significant inhibition of MNCs formation (Fig. 5). Altenuene (**8**) is a benzochromenone derivative first isolated from *Alternaria tenui*s^43^. It has potential antioxidant activity that could be attributed to its dibenzo-α-pyrone function^44^. Its ability to reduce oxidative stress can explain several of its activities such as anti-inflammatory activities^37,45^. Meanwhile, oxidative stress is directly linked to osteoclastogenesis in several ways, it can upregulate RANKL expression with the downregulation of osteoprotegerin^46,47^. This can increase osteoclast differentiation and activity. Moreover, oxidative stress can induce apoptosis of osteocytes and osteoblasts which will generate additional amounts of RANKL, promoting osteoclastogenesis^48^. Therefore, the antioxidant properties of altenuene (**8**) are believed to be the key activity explaining its anti-osteoclastogeneic potential.

### ADMET prediction

For the two active compounds (**1**,**8**), (Table S3a, Figure S9) both compounds fall within the favorable range defined by Lipinski’s Rule of Five, and neither compound shows any rule violations, indicating a high probability of good oral bioavailability and cell membrane permeability. Also, both values comply with drug-likeness criteria (HBA < 10, HBD < 5), supporting the potential for adequate membrane permeability. Compound **1** has a significantly lower water solubility (log S = −4.42) compared to compound **8** (log S = −2.23), which may pose challenges for formulation and absorption, especially for oral delivery. Both compounds have values remain below the 140 Å² threshold associated with good intestinal absorption^26^.

### ADMET properties

In terms of Caco-2 cell permeability (Table S3b), compound **1** exhibits superior performance (1.307 log Papp) over compound **8** (0.809), suggesting more efficient transcellular absorption. This is further confirmed by intestinal absorption predictions: compound **1** shows 95.08% absorption compared to 73.31% for compound **8**. Regarding blood-brain barrier (BBB) and CNS permeability, both compounds show negative log BB values (< −0.3), suggesting limited penetration into the central nervous system. This is a favorable trait for peripheral drug targets, minimizing potential CNS side effects. In terms of metabolism, compound **1** is predicted to inhibit CYP3A4, a key metabolic enzyme, which may increase the risk of drug-drug interactions. Compound **8** shows no inhibition of major cytochrome P450 enzymes, indicating a lower metabolic liability.

Clearance predictions show slightly higher total clearance (log ml/min/kg) for compound **8** (0.605) than for compound **1** (0.58), suggesting faster systemic elimination. Both compounds are predicted to be non-mutagenic (negative Ames test) and non-hepatotoxic, indicating an acceptable safety profile. However, compound **1** demonstrates a higher acute oral toxicity (LD₅₀ = 2.485 mol/kg) compared to compound **8** (LD₅₀ = 2.212 mol/kg), which could imply a narrower therapeutic window and necessitates caution in dose selection^26^.

## Conclusion

This study highlights the promising potential of sponge-derived fungi as a rich source of bioactive compounds with therapeutic applications. The identification of two fungal isolates, *A. flavus* and *C. colombiae*, underscores the importance of marine ecosystems in uncovering novel microbial resources for drug discovery. Utilizing LC-MS/MS and molecular network, eight new naturally occurring compounds (**1**, **A2**, **C1**,** C2**,** C4**-**C7**) were tentatively identified. Through bioassay-guided isolation and structural elucidation, two bioactive compounds (**1** and **8)** were discovered, demonstrating significant anti-osteoclastogenic activity by inhibiting RANKL-induced multinucleated osteoclast formation with IC_50_ values 57.14 and 38.35 µM, respectively. This suggests their potential as therapeutic candidates for the treatment of osteoporosis. Notably, based on our knowledge, this is the first report describing the anti-osteoclastogenesis of **1** and **8**. ADMET properties revealed that compound **1** demonstrates excellent predicted absorption and permeability, but poor solubility and potential CYP3A4 inhibition could limit its bioavailability and safety. While Compound **8** exhibits a better balance of solubility, safety, and metabolic stability, making it a more promising lead compound for further development as an oral drug candidate.

This study not only expands our understanding of the chemical diversity within fungi but also paves the way for the development of novel anti-osteoporotic therapies. However, further mechanistic studies are essential to elucidate the inhibitory mechanisms of these metabolites. Such insights will facilitate a comprehensive structure-activity relationship analysis and enable the development of derivatives with improved pharmacodynamic and pharmacokinetic properties.

## Electronic supplementary material

Below is the link to the electronic supplementary material.

Supplementary Material 1

## Data Availability

All materials and data are available free of charge in the manuscript and supplementary material. The datasets generated for large subunit ribosomal RNA partial sequence analysis during the current study are available in the GenBank repository, with accession numbers PQ423742 for A. flavus (Aspergillus flavus isolate B8-5 large subunit ribosomal RNA gene, part - Nucleotide - NCBI) and PQ423748 for C. colombiae (Cladosporium colombiae isolate A7-1 large subunit ribosomal RNA gene, - Nucleotide - NCBI).GenBank is a member repository of INSDC (International Nucleotide Sequence Database Collaboration).
