# Supplementary material for: Molecular networking derived from untargeted LC-MS/MS analysis to discover inhibitors of RANKL-induced osteoclastogenesis from Egyptian marine sponge-associated fungi
Source: Sci Rep. 2025 Jul 25;15:27137. doi: 10.1038/s41598-025-12456-y (PMC12297304; doi:10.1038/s41598-025-12456-y)
Supplement: Supplementary file 1 — Supplementary Material 1 [file 41598_2025_12456_MOESM1_ESM.docx]

**Molecular networking derived from untargeted LC-MS/MS analysis to discover inhibitors of RANKL-induced osteoclastogenesis from Egyptian marine sponge-associated fungi**

Abdelhalim A. Elgahamy^1^, Ahmed H. El-Desoky^2*^, Asmaa M. Otify^3*^, Ahlam M. El Fishawy^3^, Ahmed A. El-Beih^1^

^1^Chemistry of natural and microbial products Department, National Research Centre, 33 El-Bohouth St., Dokki, Giza, 12622, Egypt.

^2^Pharmacognosy Department, National Research Centre, 33 El-Bohouth St., Dokki, Giza, 12622, Egypt.

^3^Pharmacognosy Department, Faculty of Pharmacy, Cairo University, Kasr-El-Ainy, Cairo 11562, Egypt.

* Corresponding authors.

E-mail addresses: ah.el-dessouky@nrc.sci.eg (Ahmed H. El-Desoky), [asmaa.otify@pharma.cu.edu.eg](mailto:asmaa.otify@pharma.cu.edu.eg) (Asmaa M. Otify)

**Table of contents**

| Supplementary material | **Page** |
| --- | --- |
| **Table S1**: The sponge samples utilized in the study | **3** |
| Figure S1: Types and percentages of microbial isolates | **3** |
| Figure S2: Sponges harboring the two selected fungi, *A. Flavus* and *C. colombia****e*** | **3** |
| Figure S3: Macroscopical, microscopical, and large batch figures of the selected fungi (a, b, c for *A. Flavus* and d, e, f for *C. colombiae,* respectively) | **4** |
| **Figure** S4: Phylogenetic tresses for the two selected fungi (a: *Aspergillus flavus* ([PQ423742](https://www.ncbi.nlm.nih.gov/nuccore/PQ423742)), b: *Cladosporium colombiae* (PQ423748)) | **5** |
| Figure S5a: Base peak chromatograms of EtOAc fractions for *A. flavus* (a, b) and *C. colombiae* (c, d) analyzed by LC-MS/MS analysis in positive and negative mode, respectively. | **6** |
| **Table S2:** Metabolites tentatively identified in the EtOAc fractions of *A. flavus* and *C. colombiae* by LC-MS/MS | 7 |
| Figure S5b: MS/MS spectra, a, b, c, and d, of compounds A1 (*m/z* 377.1 C_21_H_28_O_6_), 2 (*m/z* 391.1), 1 (*m/z* 405.1, C_23_H_32_O_6_), and A2 (*m/z* 450.3 (ammonia adduct), C_25_H_36_O_6_), respectively, in the positive ion mode | 8 |
| Figure S5c: MS/MS spectra, a, b, c, d, e, f and g of compounds B1 (*m/z* 261.0, C_14_H_16_N_2_O_3_), 3 (*m/z* 261.0, C_14_H_16_N_2_O_3_), 7 (*m/z* 245.0, C_14_H_16_N_2_O_2_), B2 (*m/z* 311.1, C_18_H_18_N_2_O_3_), B3 (*m/z* 284.0, C_16_H_17_N_3_O_2_), B4 (*m/z* 261.1, C_15_H_20_N_2_O_2_), and B5 (*m/z* 295.1, C_18_H_18_N_2_O_2_), respectively, in the positive ion mode. | 9 |
| **F**igure S5d: MS/MS spectra, a, b, c, d, e, f, and g of compounds C1 (*m/z* 343.3, C_19_H_42_N_4_O), C2 (*m/z* 371.3, C_21_H_46_N_4_O), C3 (*m/z* 304.3, C_20_H_33_NO), C4 (*m/z* 399.3, C_23_H_50_N_4_O), C5 (*m/z* 332.3, C_22_H_37_NO), C6 (*m/z* 360.3, C_24_H_41_NO), and C7 (*m/z* 388.4, C_26_H_45_NO), respectively, in the positive ion mode. | 10 |
| **Figure S6a**: ^1^H-NMR spectrum of compound **1** (CDCl_3_, *δ* in ppm) | 11 |
| **Figure S6b**: ^13^C-NMR spectrum of compound **1** (CDCl_3_, *δ* in ppm) | 11 |
| **Figure S6c**: ^1^H-^1^H COSY spectrum of compound **1** (CDCl_3_, *δ* in ppm) | 12 |
| **Figure S6d**: HSQC spectrum of compound **1** (CDCl_3_, *δ* in ppm) | 12 |
| **Figure S6e**: HMBC spectrum of compound **1** (CDCl_3_, *δ* in ppm) | 13 |
| **Figure S7a**: ^1^H-NMR spectrum of compound **2** (CDCl_3_, *δ* in ppm) | 13 |
| **Figure S7b**: Figure S5b: ^13^C-NMR spectrum of compound **2** (CDCl_3_, *δ* in ppm) | 14 |
| **Figure S8** :^1^H-NMR spectrum of compound **8** (CD_3_OD, *δ* in ppm). | 14 |
| **Table S3a**: Physicochemical properties and drug likeness of compounds **1** and **8** by Swiss ADME | 15 |
| **Table S3b**: ADMET properties of compounds **1** and **8** by pkCMS | 15 |
| **Figure S9**: Boiled-egg plot generated by Swiss ADMET online tool for **1** and **8**. |  |

**Table S1**: The sponge samples utilized in the study

| **#** | **Sponge name** |
| --- | --- |
| **1** | *Negombata magnifica* |
| **2** | *Stylissa carteri* |
| **3** | *Siphonochalina siphonella* |
| **4** | *Irciunia strobilina* |
| **5** | *Crella cyathophora* |
| **6** | *Hemimycale arabica* |
| **7** | *Hyrtios erectus* |
| **8** | *Ircinia variabilis* |
| **9** | *Negombata sp.* |
| **10** | *Latrunculia magnifica* |

Figure S1: Types and percentages of microbial isolates

Figure S2: Sponges harboring the two selected fungi, *A. Flavus* and *C. colombiae*

Figure S3: Macroscopical, microscopical, and large batch figures of the selected fungi (a, b, c for *A. flavus* and d, e, f for *C. colombiae,* respectively)

**Molecular identification of the selected fungi:**

The selected fungi were subjected to molecular identification based on 18S rDNA.
Both fungal isolates were inoculated into 20 ml potato dextrose broth medium
individually for 3 days. Fungal mycelia were harvested using Whatman No. 1 filter
paper. The total genomic DNA was extracted using CTAB protocol (Benito *et al.*,
1993). As the fungal mycelia were dried and grinded using liquid nitrogen and
incubated at 60 °C for 1 h with CTAB buffer. chloroform/isoamyl buffer was
added and incubated in ice water bath for 10 min. Genomic DNA was purified by
isopropanol after centrifugation of the previous mixtures as previously described^1^.

DNA of the fungal isolates was amplified by polymerase
chain reaction (PCR) using internal transcribed spacer region (ITS), mainly ITS1
(5´-TCCGTAGGTGAACCTGCGG-3´) and ITS4 (5´-TCCTCCGCTTATTGAT A
TGC - 3´) designed for sequencing. The PCR amplification was carried out in 50
μl reaction mixture containing 1× PCR buffer (NEB, England), 1 nmol of dNTPs,
1 pmol of 2 mM MgSO4, 0.25 pmol of forward and reverse primers, 1 unit Taq
DNA polymerase (NEB, England) and 10 µl template DNA.
The PCR started with the following conditions: initial denaturation at 95^o^ C for
10 min and 35 cycles of denaturation at 95^o^ C for 30 s, annealing at 56^o^ C for 30 s
and elongation at 72^o^ C for 60 s with final extension at 72^o^ C for 10 min. Agarose
gel (1% in 1X TBE buffer) was used to electrophorese PCR products . The gel was visualized under UV light (254 nm). The PCR product is
purified by QIA quick Gel Extraction Kit (QIAGEN, USA) according to the
manufactures. The sequencing for PCR product was occurred in GATC Company
by use ABI 3730 × DNA sequencer (Germany) by using forward and reverse
primers. The identification was achieved by comparing the contiguous DNA
sequence with data from the reference and type strains available in public databases
GenBank using the BLAST program (National Centre for Biotechnology
Information) (http://www.ncbi.nlm.nih.Gov /BLAST). The obtained sequences
were aligned using Jukes Cantor Model. Phylogenetic analysis was carried out by
the neighbor-joining method using mega software ^2^

**Figure** S4: Phylogenetic tresses for the two selected fungi

(a: *Aspergillus flavus* ([PQ423742](https://www.ncbi.nlm.nih.gov/nuccore/PQ423742)), b: *Cladosporium colombiae* (PQ423748))

Figure S5a: Base peak chromatograms of EtOAc fractions for *A. flavus* (a, b) and *C. colombiae* (c, d) analyzed by LC-MS/MS analysis in positive and negative ion modes, respectively.

**Table S2:** Metabolites tentatively identified in the EtOAc fractions of *A. flavus* and *C. colombiae* by LC-MS/MS.

| ***#*** | **Rt**  **(min)** | ***m/z*** | **Molecular formula** | ***MS/MS*** | **Compound** |
| --- | --- | --- | --- | --- | --- |
| **Bisphenols** | | | | | |
| **Al** | 5.8 | 377.1 | C_21_H_28_O_6_ | **209**, 191, 135 | 2,2-bis-[4-(2,3-Dihydroxy-propoxy)phenyl]propane |
| **2** | 7.8 | 391.1 | C_22_H_30_O_6_ | 223, **209**, 135 | 2‐[4‐(2-Hydroxy-3‐methoxy-propoxy)phenyl]‐2‐[4‐(2,3-dihydroxy-propoxy)phenyl]propane |
| **1** | 9.8 | 405.1 | C_23_H_32_O_6_ | **223**, 191, 135 | 2,2‐bis‐[4‐(2-Hydroxy-3-methoxy-propoxy)phenyl]propane |
| **A2** | 12.7 | 450.3^a^ | C_25_H_36_O_6_ | 391, 223, **209** | 2-[4-(2-hydroxy-3-methoxy-propoxy)phenyl]-2-[4-(2-hydroxy-3-propoxy-propoxy)phenyl]propane |
| **Diketopiperazines** | | | | | |
| **B1** | 1.6 | 261.0 | C_14_H_16_N_2_O_3_ | 245, 233, **170** | cyclo(Prolyl-tyrosyl) |
| **3** | 2.1 | 261.0 | C_14_H_16_N_2_O_3_ | 233, 188, **120** | *3S*,6S*,8R**-cyclo(*trans*-8-Hydroxyprolyl-phenylalanyl) |
| **7** | 2.9 | 245.0 | C_14_H_16_N_2_O_2_ | 217, 172, **120** | *3S*,6S**-cyclo(Prolyl-phenylalanyl) |
| **B2** | 3.1 | 311.1 | C_18_H_18_N_2_O_3_ | **283**, 205, 136 | cyclo(Phenylalanyl-tyrosyl) |
| **B3** | 3.3 | 284.0 | C_16_H_17_N_3_O_2_ | 267, 170, **130** | cyclo(Prolyl-tryptophanyl) |
| **B4** | 4.6 | 261.1 | C_15_H_20_N_2_O_2_ | **233**, 216**,** 120 | cyclo(Leucyl-phenylalanyl) |
| **B5** | 5.1 | 295.1 | C_18_H_18_N_2_O_2_ | **267**, 250, 120 | cyclo(Phenylalanyl-phenylalanyl) |
| **Pyrrolidines** | | | | | |
| **C1** | 9.7 | 343.3 | C_19_H_42_N_4_O | **240** | 2-(3-Diaminomethyl-aminopropyl)-3-hydroxyl-5-undecyl-pyrrolidine |
| **C2** | 12.5 | 371.3 | C_21_H_46_N_4_O | **268** | 2-(3-Diaminomethyl-aminopropyl)-3-hydroxyl-5-tridecyl-pyrrolidine |
| **C3** | 13.7 | 304.3 | C_20_H_33_NO | **212** | 2-Benzyl-3-hydroxy-5-nonyl-pyrrolidine |
| **C4** | 15.4 | 399.3 | C23H_50_N_4_O | **296** | 2-(3-Diaminomethyl-aminopropyl)-3-hydroxyl-5-pentadecyl-pyrrolidine |
| **C5** | 16.3 | 332.3 | C_22_H_37_NO | **240** | 2-Benzyl-3-hydroxyl-5-undecyl-pyrrolidine |
| **C6** | 17.3 | 360.3 | C_24_H_41_NO | **268** | 2-Benzyl-3-hydroxyl-5-tridecyl-pyrrolidine |
| **C7** | 20.0 | 388.4 | C_26_H_45_NO | **296** | 2-Benzyl-3-hydroxyl-5-pentadecyl-pyrrolidine |

^a^ Corresponds to the ammonia adduct; numbers in bold represent the base peak, * corresponds to relative configuration

Figure S5b: MS/MS spectra, a, b, c, and d, of compounds A1 (*m/z* 377.1 C_21_H_28_O_6_), 2 (*m/z* 391.1), 1 (*m/z* 405.1, C_23_H_32_O_6_), and A2 (*m/z* 450.3 (ammonia adduct), C_25_H_36_O_6_), respectively, in the positive ion mode.

Figure S5c: MS/MS spectra, a, b, c, d, e, f and g of compounds B1 (*m/z* 261.0, C_14_H_16_N_2_O_3_), 3 (*m/z* 261.0, C_14_H_16_N_2_O_3_), 7 (*m/z* 245.0, C_14_H_16_N_2_O_2_), B2 (*m/z* 311.1, C_18_H_18_N_2_O_3_), B3 (*m/z* 284.0, C_16_H_17_N_3_O_2_), B4 (*m/z* 261.1, C_15_H_20_N_2_O_2_), and B5 (*m/z* 295.1, C_18_H_18_N_2_O_2_), respectively, in the positive ion mode.

Figure S5d: MS/MS spectra, a, b, c, d, e, f, and g of compounds C1 (*m/z* 343.3, C_19_H_42_N_4_O), C2 (*m/z* 371.3, C_21_H_46_N_4_O), C3 (*m/z* 304.3, C_20_H_33_NO), C4 (*m/z* 399.3, C_23_H_50_N_4_O), C5 (*m/z* 332.3, C_22_H_37_NO), C6 (*m/z* 360.3, C_24_H_41_NO), and C7 (*m/z* 388.4, C_26_H_45_NO), respectively, in the positive ion mode.


**Figure S6a**: ^1^H-NMR spectrum of compound **1** (CDCl_3_, *δ* in ppm).

**Figure S6b**: ^13^C-NMR spectrum of compound **1** (CDCl_3_, *δ* in ppm).

**Figure S6c**: ^1^H-^1^H COSY spectrum of compound **1** (CDCl_3_, *δ* in ppm)

**Figure S6d**: HSQC spectrum of compound **1** (CDCl_3_, *δ* in ppm).

**Figure S6e**: HMBC spectrum of compound **1** (CDCl_3_, *δ* in ppm).

**Figure S7a** :^1^H-NMR spectrum of compound **2** (CDCl_3_, *δ* in ppm).

**Figure S7b**: ^13^C-NMR spectrum of compound **2** (CDCl_3_, *δ* in ppm).

**Figure S8** :^1^H-NMR spectrum of compound **8** (CD_3_OD, *δ* in ppm).

**Table S3a:** Physicochemical properties and drug likeness of compounds **1** and **8** by Swiss ADME

| **Compound** | **MW** | **nHA** | **HBA** | **HBD** | **MR** | **TPSA** | **RB** | **Lipophilicity**  **Log PO/W** | **Water solubility**  **Log S** | **Drug likeness**  **Lipinski’s rule**  **of 5** |
| --- | --- | --- | --- | --- | --- | --- | --- | --- | --- | --- |
| **1** | 404.50 | 29 | 6 | 2 | 111.71 | 77.38 | 12 | 3.12 | -4.42 | Yes; 0 violation |
| **8** | 292.28 | 21 | 6 | 3 | 73.69 | 96.22 | 1 | 1.03 | -2.23 | Yes; 0 violation |

Abbreviations: MW = Molecular weight (Dalton); nHA = Number of heavy atoms; HBA =Number of hydrogen bond acceptors; HBD = Number of hydrogen bond donors; MR = Molecular refractivity; TPSA =Topological polar surface area (Å^2^); RB = Number of rotatable bonds.

**Table S3b**: ADMET properties of compounds **1** and **8** by pkCMS

| **Compound**  **code** | **Absorption** | | | **Distribution** | | **Metabolism** | | **Excretion** | **Toxicity** | | | |
| --- | --- | --- | --- | --- | --- | --- | --- | --- | --- | --- | --- | --- |
|  | **Caco-2** | **Int. abs** | **P-gp** | **BBB** | **CNS** | **CYP2D6** | **CYP3A4** | **TC** | **Ames** | **MRTD** | **LD50** | **Htox** |
| **1** | 1.307 | 95.083 | I | -0.697 | -3.331 | No | Yes | 0.58 | No | 0.6 | 2.485 | No |
| **8** | 0.809 | 73.308 | 0 | -0.731 | -3.388 | No | No | 0.605 | No | 0.252 | 2.212 | No |

Abbreviations: Caco-2 permeability (log Papp in 10-6 cm/s); Int. abs = Intestinal absorption (% Absorbed); P-gp = P-glycoprotein inhibitors I or II; BBB permeability = Blood-brain barrier permeability (log BB); CNS permeability = Central nervous system permeability (log PS); TC = Total clearance (log ml/min/kg); MRTD =Maximum tolerated dose (human) (log mg/kg/day); LD50 = Lethal Dose at 50% Inhibition (Oral Rat Acute Toxicity (mol/kg)) ; Htox= Hepatotoxicity.

**
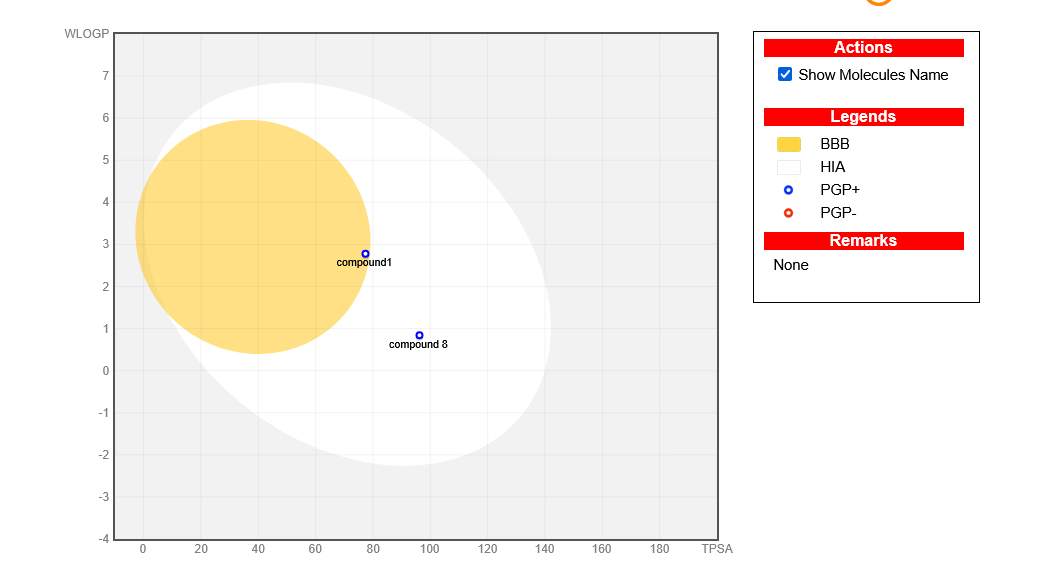
**

**Figure S9**: Boiled-egg plot generated by Swiss ADMET online tool for **1** and **8**.
